# Tactical tuning of the surface and interfacial properties of graphene: A Versatile and rational electrochemical approach

**DOI:** 10.1038/s41598-017-08627-1

**Published:** 2017-08-21

**Authors:** Chiranjeevi Srinivasa Rao Vusa, Manju Venkatesan, Aneesh K, Sheela Berchmans, Palaniappan Arumugam

**Affiliations:** Council of Scientific and Industrial Research- Central Electrochemical Research Institute, Karaikudi, 630003 India

## Abstract

Designing a versatile and rational method for the tactical tuning of the surface and interfacial properties of graphene is an essential yet challenging task of many scientific areas including health care, sensors, energy, and the environment. A method was designed herein to tackle the challenge and tune the surface and interfacial properties of graphene using a simple electrochemical tethering of arylamines that provides diverse reactive end groups to graphene. This method resulted in the preparation of graphenes with thiol, hydroxy, amine, carboxyl, and sulfonate surface functionalities respectively. X-ray photoelectron spectroscopy, scanning electron microscopy, and cyclic voltammetry were used to study the chemical, morphological, and electrochemical properties of the modified graphenes. The results show the promising scope of the reported method towards the tactical tuning of the surface and interfacial properties of graphene. Also, this method can give fundamental insights of the surface tuning of graphene and its structurally similar materials. Hence, this approach can be used to advantageously tune the surface properties of the other structurally similar nanocarbons and their hybrid materials to make them potential candidates for many applications.

## Introduction

In recent times, the material graphene is being considered as an obvious alternative of carbon allotropes^1^ in the fields of sensors, energy conversion devices^1^, transparent electrodes^2^, nanoelectronics^3^, and photonics^2^. Very often tuning of the surface and interfacial properties of graphene become a prerequisite to making the material an effective candidate for the given application^4–6^. In general, researchers have been adopting protocols similar to that employed for the covalent functionalization of its allotropes graphite, fullerene and CNTs^7, 8^. Most of these methodologies which employ aryl diazonium species are spontaneous on metals and semiconductors than on the graphene^9–11^. Hence, the procedures may not be suitable for the applications that require direct tuning of graphene in the case of lab on chip devices, chemical and biosensor arrays. The issue of controllable and versatile tuning of the surface properties of graphene is still a great challenge to the research community. The tuning of carbon surfaces by electrochemical methods is more efficient than other procedures as these routes are simple to adopt, require lesser time and can be performed under eco-friendly conditions^12–14^. The electrochemical tethering of amines to carbon surface reported by Blandine Barbier has been utilized herein^12^.

**Figure 1:**
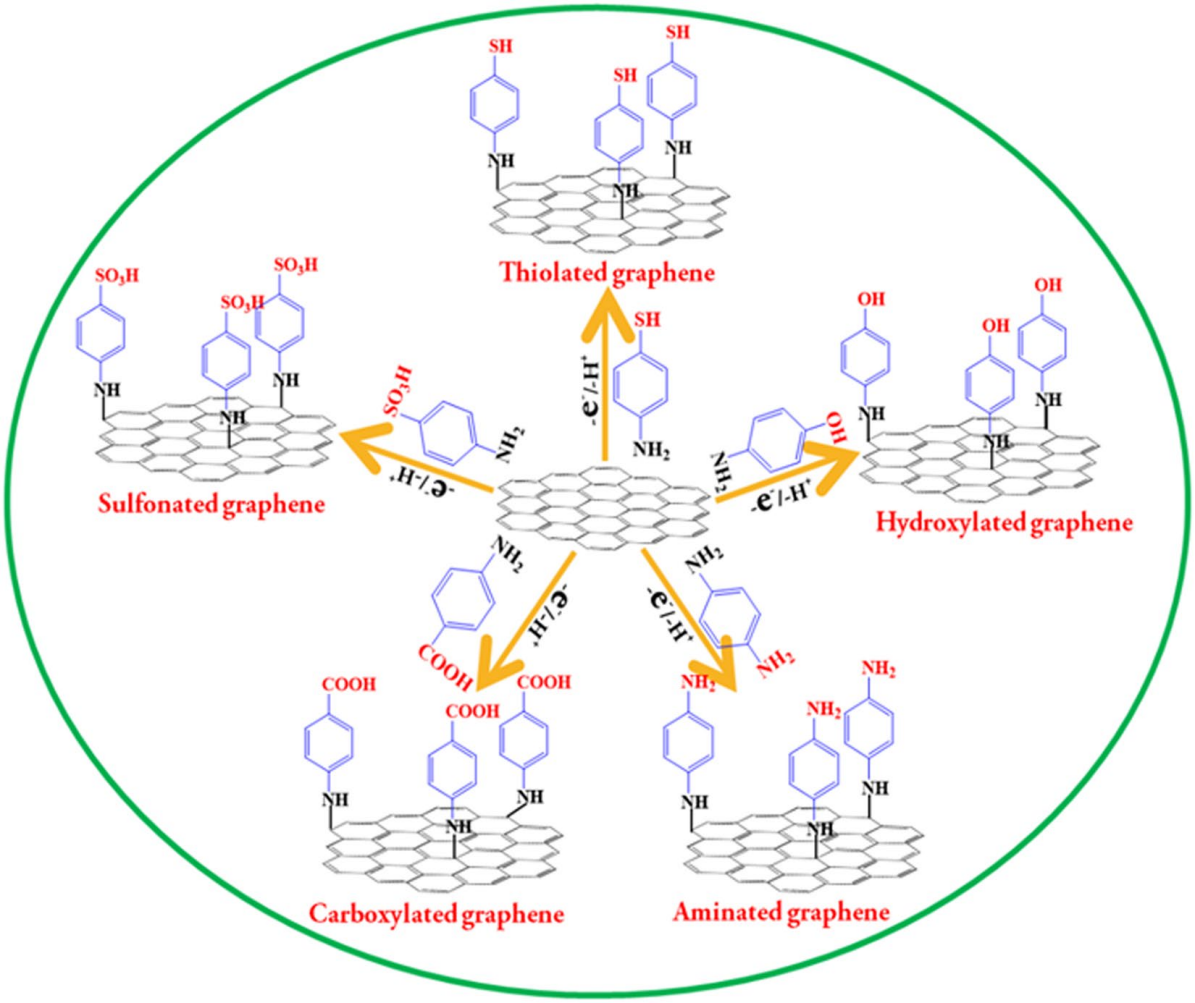
The schematic representation of electrochemical functionalization of graphene.

Herein, a versatile and rational electrochemical method has been designed for the tactical tuning of the surface and interfacial properties of graphene by covalently linking arylamine derivatives containing different reactive terminal groups (-SH, -OH, -NH_2_, -COOH and -SO_3_H). The five arylamine derivatives utilized herein are aminothiophenol (ATP), aminophenol (AP), para-phenylenediamine (PPDA), aminobenzoic acid (ABA) and sulfanilic acid (SA) which possess the reactive end groups -SH, -OH, -NH_2_, -COOH and -SO_3_H respectively. The amine functional group present in the corresponding derivative is electro-oxidized to form a covalent bond between amine and graphene yielding graphenes with thiol, hydroxy, amine, carboxyl, and sulfonate surface functionalities. The anodic oxidation of primary amine forms an amine radical cation that loses a proton and forms an amine radical. Then, the formed amine radical attacks the sp^2^ carbon of the graphene and forms a C-N covalent bond between the amine and graphene. This mechanism has been well established with the other allotropes of carbon including fullerenes, carbon nanotubes, carbon fibers, graphite, etc^12–15^. This versatile protocol leads to the formation of a stable and compact monolayer of the modifier molecule on the graphene surface. The surface decorated graphene thus obtained would be of great interest to researchers of chemical sensors, biosensors, immunosensors, and DNA sensors for tethering enzymes, redox mediators, DNA, proteins, antigens, and antibodies^16^. The surface tuned graphenes thus prepared are characterized by x-ray photoelectron spectroscopy (XPS), field emission scanning electron microscopy (FE-SEM) and cyclic voltammetry (CV). The interfacial electron transfer properties of the graphenes are investigated by cyclic voltammetry using a positively charged, [Ru(NH_3_)_6_]Cl_3_, and a negatively charged, K_4_[Fe(CN)_6_], redox system. Besides, the electrochemical behavior of two essential bioanalytes dopamine (DA) and ascorbic acid (AA) is also investigated. The above studies provide the fundamental insights about the influence of the terminal functional groups in tuning the surface and interfacial properties of graphene. Figure 1 shows the tactical tuning of the surface properties of graphene by versatile and rational electrochemical method.

## Experimental section

### Reagents and solutions

Graphite powder (≤20 µM), α-Al_2_O_3_, potassium permanganate, sodium nitrate, hydrogen peroxide (H_2_O_2_), LiClO_4_, potassium chloride, sodium hydroxide, ruthenium hexamine chloride, dopamine, p-aminothiophenol, p-aminophenol, p-phenylenediamine, p-aminobenzoic acid and sulfanilic acid were purchased from Sigma-Aldrich. Sulfuric acid was purchased from Fisher-Scientific. Potassium ferrocyanide and potassium hydrogen phosphate were purchased from Hi-Media. Acetonitrile was purchased from SRL. All the chemicals were used as received without any purification. Ultrapure water from Siemens LaboStar (resistivity ~18.2 MΩ.cm) system was used to prepare phosphate buffer and other aqueous solutions.

### Apparatus

All the electrochemical experiments were carried out using Autolab PGSTAT-302N from Metrohm. A three electrode system consisting of glassy carbon electrode (GCE, 3 mm diameter) or surface tuned graphene modified glassy carbon electrode as a working electrode, spiral platinum wire as a counter electrode and normal calomel electrode (NCE) as a reference electrode was used. A silver wire was used as a pseudo reference electrode in non-aqueous solution based electrochemical experiments. Cyclic voltammograms were recorded using (1) negatively charged redox probe 1 mM K_4_[Fe(CN)_6_] in 1 M KCl and (2) positively charged redox probe 1 mM [Ru(NH_3_)_6_]Cl_3_ in 1 M KCl to get the fundamental insights and electrochemical properties of the surface tuned graphenes. Also, the voltammetric response of two bioanalytes dopamine (a positively charged bioanalyte) and ascorbic acid (negatively charged bioanalyte) were studied to check the feasibility of the surface tuned graphenes towards bioanalytical applications. The changes in peak current and peak-to-peak separations were tracked to evaluate the electrochemical properties of the surface tuned graphenes in the above cases. All the electrochemical experiments were carried out at room temperature and in dissolved O_2_ condition with a stationary working electrode.

All the XPS studies were done with MULTILAB 2000 based X-Ray photoelectron spectrometer using monochromatic Al K_α_ X-Ray source operated at 150 W and the spectra were deconvoluted by CASA XPS using Shirley baseline with a combination of 20% Gaussian and 80% Lorentzian function. In all the cases, the secondary electron background was subtracted using the Shirley function. Also, the XPS spectra were referenced to the C1s peak from the adventitious carbon at 284.8 eV to eliminate any positive charge-induced binding energy shift. All the topographical studies were done using FE-SEM Zeiss Supra 55VP from CARL ZEISS equipped with a high brightness conical FE gun.

### Synthesis of graphene from graphene oxide

Hummer’s method was adopted to synthesize graphene oxide (GO)^17^. Graphene (Gr) was synthesized using the previously reported method by our team^18^. In brief, carrot extract was obtained by boiling 20 g of carrot in 100 mL deionized water at 100 °C for 50 min, then filtered using whatman-40 filter paper. Then the 100 mL of the carrot extract was added to the flask containing 20 mL of GO (1 mg/mL) and 200 mL of 1 M NaOH. The resulting solution was stirred for 2 h at 90 °C. The black suspension obtained was filtered, centrifuged and washed with water for 5 times. Finally, the obtained black precipitate was lyophilized to get the graphene powder.

### Preparation of graphene modified GCE

Graphene suspension was prepared by dispersing 5 mg in 5 mL DMF for two hours by ultrasonication. Before the covalent functionalization, GCE was polished using 0.05 µm α-Al_2_O_3_ and abrasive papers and then sonicated in alcohol and double distilled water respectively. Then, 10 µl of the graphene dispersion (1 mg/mL) was drop casted on the cleaned GCE and allowed to dry at room temperature for 3 h.

### Tuning of the surface properties of graphene by electrochemical method using arylamine derivatives

Four graphene modified GCEs were scanned from 0.0 V to 1.0 V in aqueous solutions of 0.1 M LiClO_4_ containing 5 mM of AP (for Gr-OH), PPDA (for Gr-NH_2_), ABA (for Gr-COOH), and SA (for Gr-SO_3_H) separately for five cycles at a scan rate of 20 mVs^−1^ by cyclic voltammetry to obtain the surface tuned graphenes. As ATP is water insoluble, thiolation of graphene (Gr-SH) was done in an acetonitrile solution of 0.1 M LiClO_4_ containing 5 mM ATP by the procedure mentioned above.

## Results and Discussion

**Figure 2:**
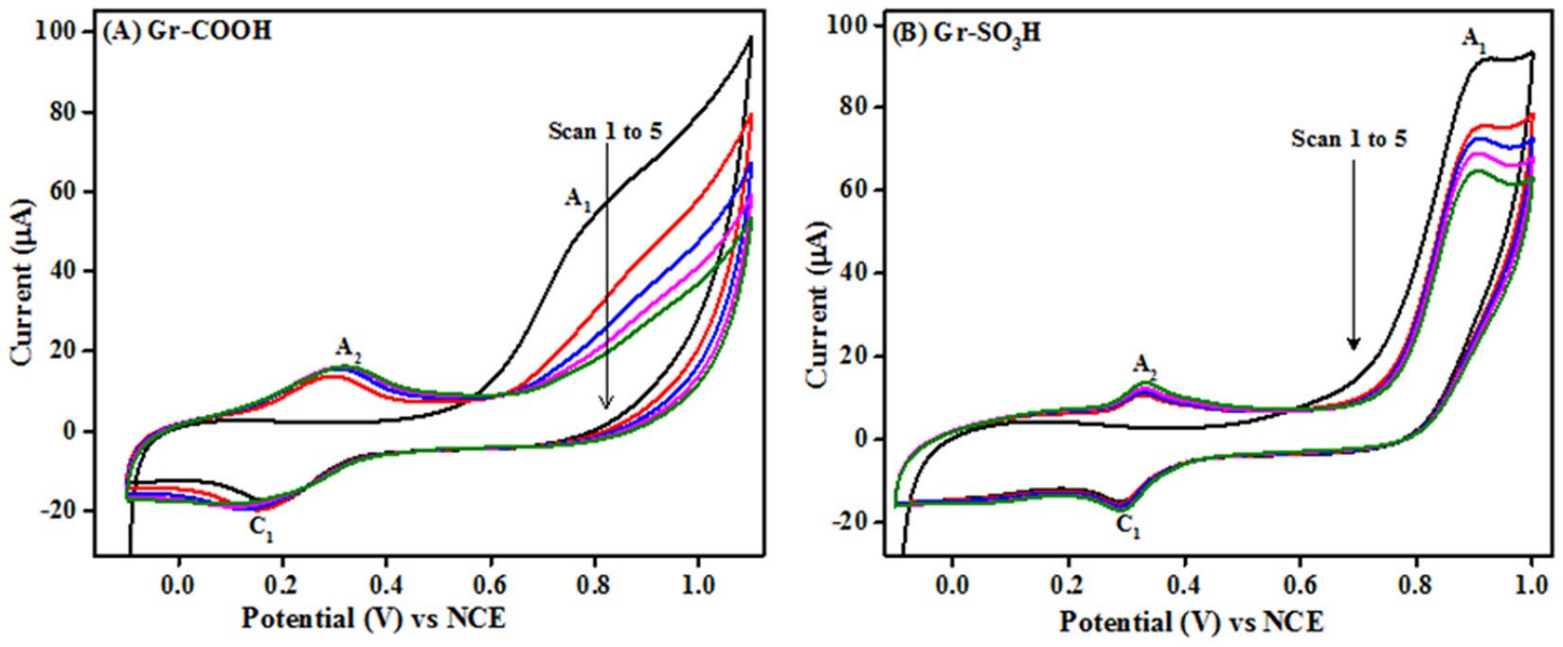
The cyclic voltammograms of electrochemical (**A**) carboxylation and (**B**) sulfonation of graphene in 0.1 M LiClO_4_ containing ABA and SA respectively.

**Figure 3:**
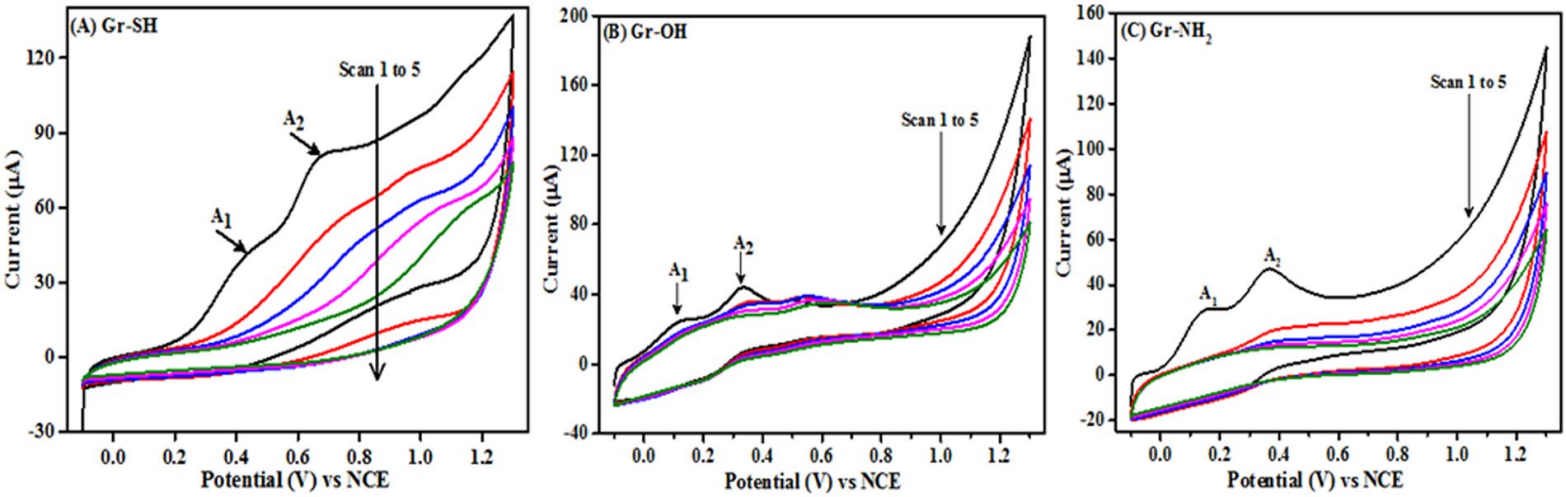
The cyclic voltammograms of electrochemical (**A**) thiolation, (**B**) hydroxylation and (**C**) amination of graphene in 0.1 M LiClO_4_ containing ATP, AP, and PPDA respectively.

Electrochemical oxidation of arylamines at graphene surface tuned the surface and interfacial properties of graphene by tethering different reactive end groups -SH, -OH, -NH_2_, -COOH and -SO_3_H to the graphene surface using cyclic voltammetry. Figures 2 and 3 show the cyclic voltammograms recorded during the surface tuning of graphene with the arylamines having electron withdrawing (-COOH, -SO_3_H) and electron donating (-SH, -OH, -NH_2_) reactive end groups respectively. The irreversible peak A_1_ appearing in Figs 2 and 3 represent the formation of corresponding amine cation radical during one electron electro-oxidation. The so formed amine cation radical rapidly loses a proton and forms an amine radical, which then attacks the sp^2^ carbon of graphene forming a covalent bond between amine and graphene^12, 13, 15^. It is worth noting that the potential required to generate the corresponding amine cation radical of the arylamine derivative depends on the electron withdrawing tendency of the reactive end group present. The Epa (of A_1_) of the arylamine derivatives is as follows 125 mV (for -OH substituted) < 145 mV (for -NH_2_ substituted) < 815 mV (for –SH substituted) < 830 mV (for -COOH substituted) < 905 mV (for -SO_3_H substituted). This trend might be explained by the availability of electrons at amine group. i.e. the more the electron donating tendency of the reactive end group, the more will be the availability of the electrons at amine group of the molecule, which leads to the easy oxidation of the amine functional group of the molecule at lower overpotential.

As shown in Fig. 2, the reversible wave (A_2_ and C_1_) might be attributed to the formation of the head to tail type aminodiphenylamine dimer, which is known for its redox activity during this kind of electrochemical cycling process^19^. The second oxidation peak A_2_ appearing in Fig. 3A,B and C is due to the formation of dication radical from the oxidation of amine monocation radical of the respective arylamine derivative^20^. From Figs 2 and 3, the decrease or disappearance of the oxidation peak A_1_ in successive cycles represents the formation of an irreversible layer of the corresponding arylamine derivative on graphene. Hence, the layer formed either stop the oxidation process or kinetically decelerates the electron transfer as a function of time^21^. The density of molecules bonded to graphene in each case was calculated from the charge consumed in the first anodic sweep of the corresponding molecule using the equation (1) ^13, 22^

1 $${\boldsymbol{Q}}={\boldsymbol{nFA}}\frac{{\boldsymbol{\Gamma }}}{{{\boldsymbol{N}}}_{{\boldsymbol{A}}}}$$

where Q is the area under the A_1_ (background subtracted), n is the number of electrons involved (n = 1), F is the Faraday constant, A is the effective surface area of the graphene modified GCE (0.1104 cm^−2^, calculated by Randles-Sevcik equation), N_A_ is the Avogadro number and Γ is surface density of the molecules attached on graphene assuming that all the radicals generated in the first anodic scan were attached to graphene. The density of the aryl amines substituted with the terminal functionalities -SH, -OH, -NH_2_, -COOH and -SO_3_H respectively on graphenes are 45.23 × 10^15^ molecules cm^−2^, 38.43 × 10^15^ molecules cm^−2^, 40.99 × 10^15^ molecules cm^−2^, 44.1 × 10^15^ molecules cm^−2^ and 39.68 × 10^15^ molecules cm^−2^, which is higher than the reported values with aryl diazonium salts^6, 22^. The standard deviation of the surface density of molecules bonded to graphene is found to be ±3 for different modifications. The slight differences in their molecular densities might be due to the different chemistry (stearic effects, the ease of radical formation, the stability of the radical generated, the chemistry between the radical and graphene) of the reactive end group of the arylamines and their interactions with graphene during the surface tuning.

### Spectroscopic characterization of the surface tuned graphenes

**Figure 4:**
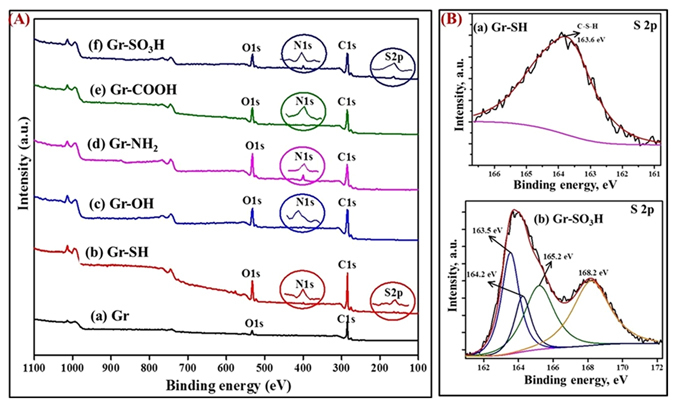
(**A**) XPS of (a) graphene, (b) thiolated graphene, (c) hydroxylated graphene, (d) aminated graphene, (e) carboxylated graphene and (f) sulfonated graphene. (**B**) The high resolution S2p XPS spectra of (a) thiolated graphene and (c) sulfonated graphene. The circled parts of the spectra are the magnified views of N1s and S2p regions.

XPS characterization is one of the most reliable techniques to analyze aryl layers bonded to graphene. As shown in the survey spectra of graphene and surface tuned graphenes in Fig. 4A, the appearance of an N1s peak at 400 ± 3 eV after the surface tuning of graphene by arylamine derivatives manifests the presence of nitrogen in the surface tuned graphenes. The peaks centered at 399.3 ± 0.3 and 400.5 ± 0.5 eV in the deconvolution spectra of N1s in the surface tuned graphenes approve the presence of (sp^2^) C-N and (sp^3^) C-N covalent bonds^23^. The (sp^2^) C-N and (sp^3^) C-N bonds could be between the aryl carbon and nitrogen from the same arylamine derivative and sp^3^ carbon of the graphene and the nitrogen of the arylamine respectively. The same (sp^3^) C-N covalent bond has been reflected again by the peak centered at 287.0 ± 1 eV in the deconvolution of spectra of C1s of the surface tuned graphenes^23–25^ shown in the supporting information Figs S1 and S2.

**Figure 5:**
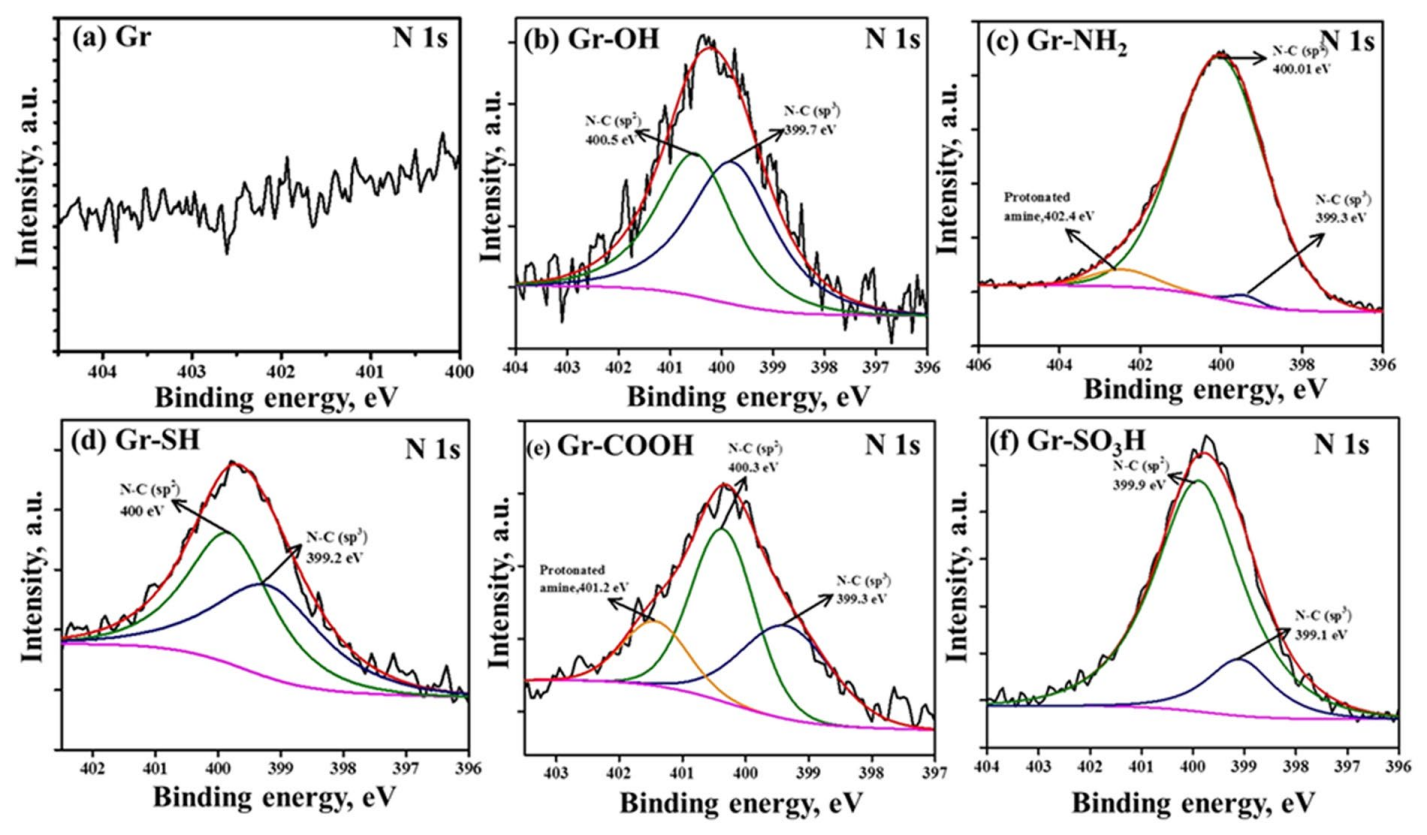
The high-resolution N1s XPS spectrum of (**a**) graphene, (**b**) hydroxylated graphene, (**c**) aminated graphene, (**d**) thiolated graphene, (**e**) carboxylated graphene and (**c**) sulfonated graphene.

The deconvoluted high-resolution N1s spectrum of Gr-NH_2_ demonstrates the presence of -C-N and protonated amine on the surfaces, as illustrated in Fig. 5. This observation again confirms the effective amination of graphene by the electrochemical protocol. The presence of sulfur functionality in the graphenes having thiol and sulfonate surface functionality has been verified by the S2p peak appearing at 164 eV in survey and deconvolution spectra of the respective graphenes^26, 27^ as shown in the Fig. 4A,B. The survey spectra of Gr-OH and Gr-COOH and the deconvolution spectra of C1s and O1s in Figs 4A and S2 confirm the signatures of -OH and -COOH functionality in the graphenes having hydroxyl and carboxylate functionality^28–30^. The surface elemental composition of all the surface tuned graphenes and the relative distribution of sp^3^ C-N, sp^2^ C-N and protonated amines present in the same are tabulated in Tables S1 and S2 respectively in the supporting information for better understanding. Thus, the XPS analysis results confirm the effective tuning of the surface of the graphenes with the thiol, hydroxyl, amine, carboxyl, and sulfonate functionality by the rational electrochemical method.

### The morphological characterization of graphene and surface tuned graphene

**Figure 6:**
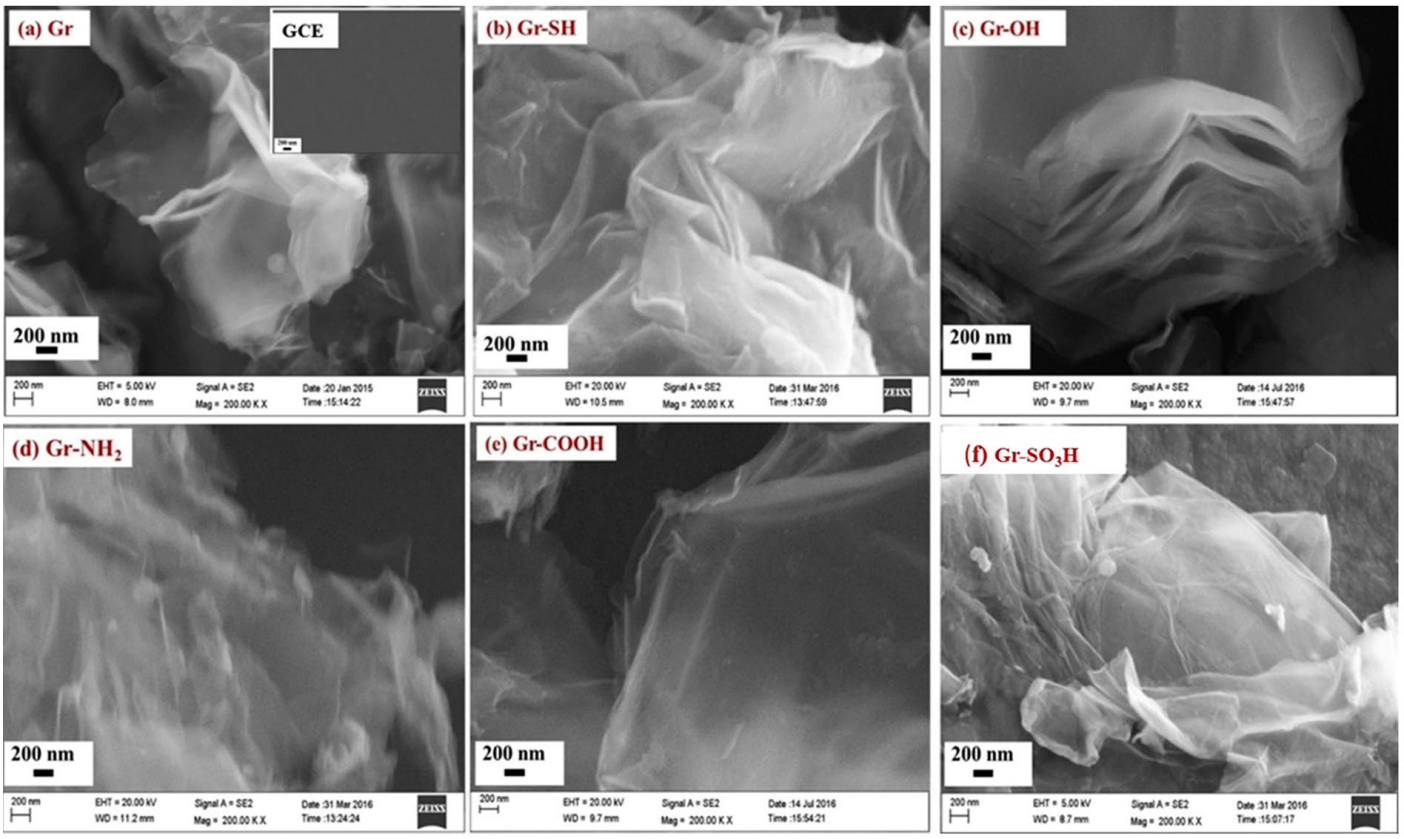
FE-SEM images of (**a**) graphene (inset shows GCE), (**b**) thiolated graphene, (**c**) hydroxylated graphene, (**d**) aminated graphene, (**e**) carboxylated graphene and (**c**) sulfonated graphene.

The topography of the graphene before and after the surface tuning with the arylamine derivatives is scrutinized by FE-SEM as shown in Fig. 6. The GCE shows a smooth and glassy surface whereas the crumpled and wrinkled morphology is observed on graphene modified GCEs which is a typical morphology of graphene reported by researchers, as shown in Fig. 6a. After surface tuning, there is no significant change in the surface morphology of the graphenes and no polymer signatures on the surface tuned graphenes as well, as shown in Fig. 6b to f. This observation clearly substantiates that molecular level tethering of the modifier molecules on the graphenes has been achieved by the electrochemical grafting procedure. The above mentioned molecular level tuning of the carbon surfaces is of great significance in micro and nanoelectrode surface engineering in a variety of applications^31^.

### Electrochemical properties of surface tuned graphene

**Figure 7:**
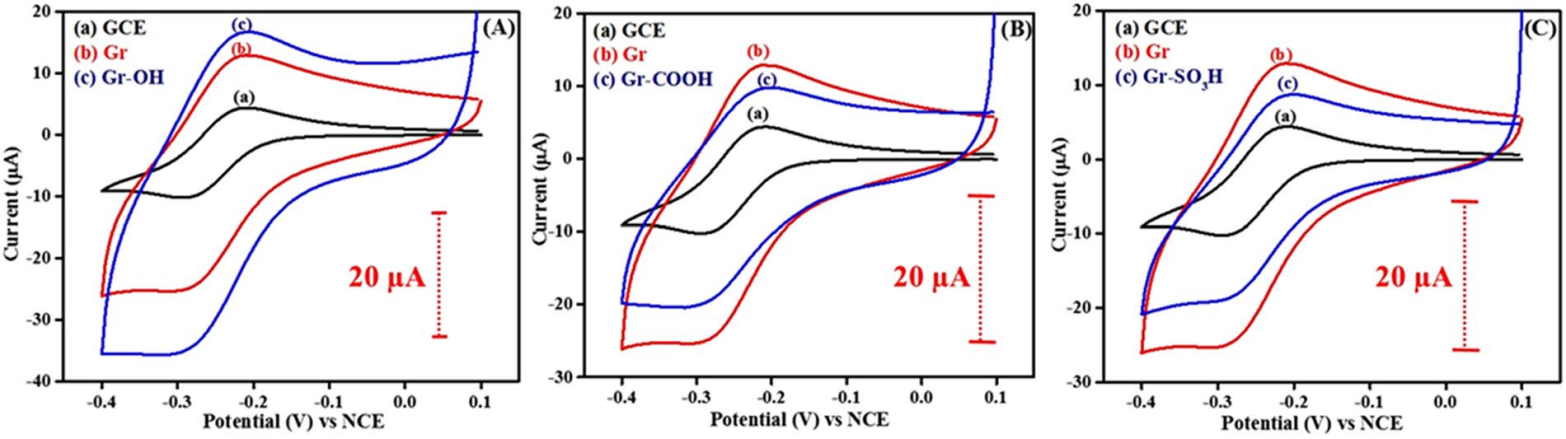
Cyclic voltammograms of (a) GCE, (b) Gr, (c) in (**A**) Gr-OH, (**B**) Gr-COOH and (**C**) Gr-SO_3_H recorded in 1 M KCl solution containing 1 mM [Ru(NH_3_)_6_]Cl_3_ at 50 mV s^−1^. The dotted bars represent the magnitude of current scale.

Cyclic voltammetry studies were carried out with a positively and negatively charged redox probes, [Ru(NH_3_)_6_]Cl_3_ and K_4_[Fe(CN)_6_] respectively to get the fundamental insights of the interfacial properties of the surface tuned graphenes. Figure 7 shows the cyclic voltammograms of [Ru(NH_3_)_6_]Cl_3_ on Gr-OH, Gr-COOH, and Gr-SO_3_H. The peak potential difference (ΔE_p_) values of the positively charged redox probe, [Ru(NH_3_)_6_]Cl_3_ on Gr-OH (74 mV), Gr-COOH (85 mV) and Gr-SO_3_H (83 mV) do not significantly deviate from that of untuned graphene (78 mV). The behavior is a common observation in the case of outer sphere redox probes like [Ru(NH_3_)_6_]Cl_3_. The cathodic or anodic peak currents observed for the [Ru(NH_3_)_6_]Cl_3_ are in the order GCE < Gr-SO_3_H < Gr-COOH < Gr < Gr-OH. The higher currents realized at Gr and Gr-OH might be attributed to the high surface area, higher conductivity of Gr and some charge based interactions between the redox probe and the Gr-OH. The lower currents observed at Gr-SO_3_H and Gr-COOH might be due to the high molecular density and steric hindrance exhibited by the large reactive end groups of the aryl layer present on the Gr-SO_3_H and Gr-COOH.

**Figure 8:**
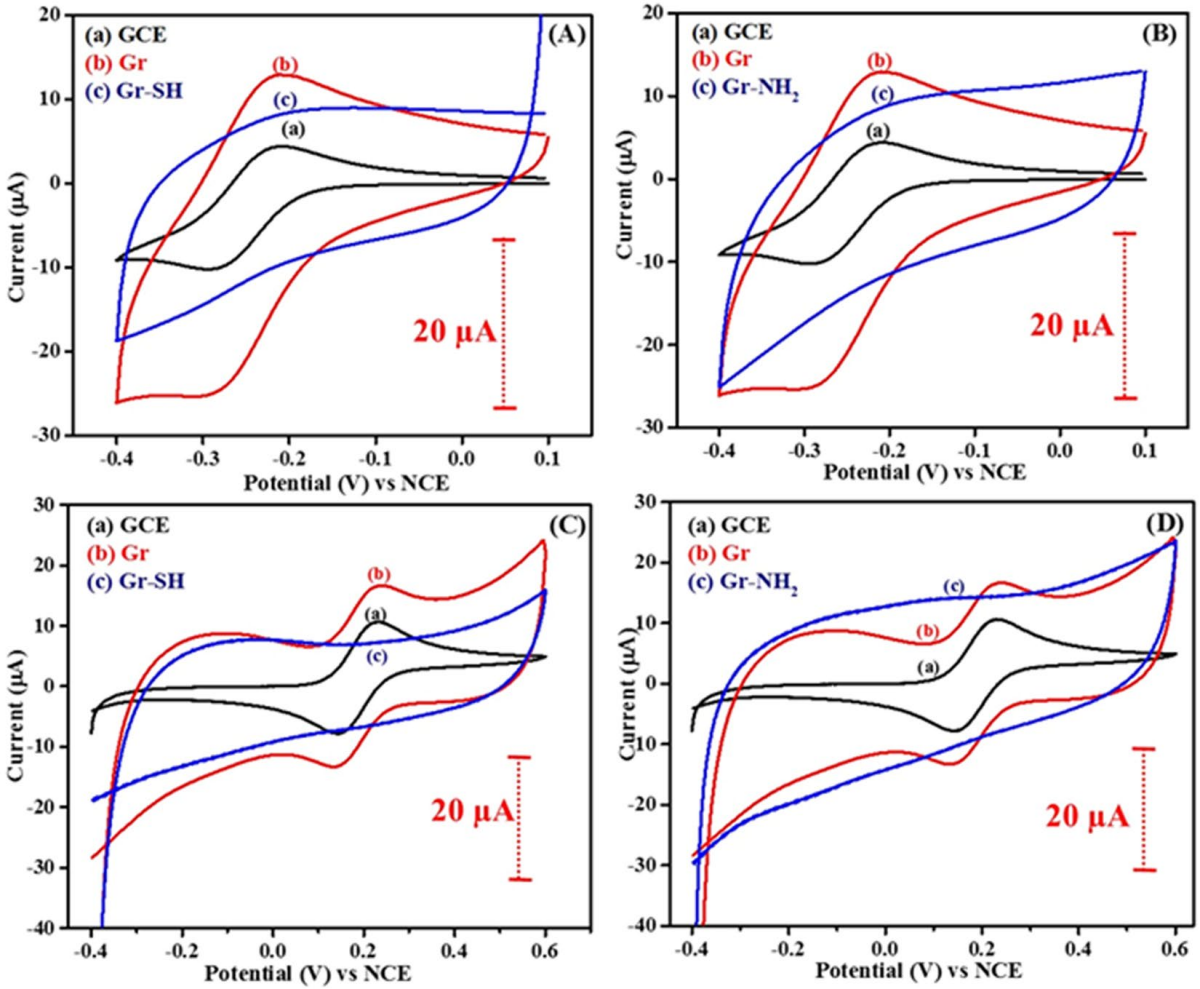
Cyclic voltammograms of (a) GCE, (b) Gr, (c) in (**A**) Gr-SH and (**B**) Gr-NH_2_ recorded in 1 M KCl solution containing 1 mM [Ru(NH_3_)_6_]Cl_3_ at 50 mV s^−1^. Cyclic voltammograms of (a) GCE, (b) Gr, (c) in (**C**) Gr-SH and (**D**) Gr-NH_2_ recorded in 1 M KCl solution containing 1 mM K_4_[Fe(CN)_6_] at 50 mV s^−1^. The dotted bars represent the magnitude of current scale.

However, to the contrary, the redox response of [Ru(NH_3_)_6_]Cl_3_ is completely blocked on Gr-SH and Gr-NH_2_ (shown in Fig. 8A,B). This behavior might be attributed to the poor conductivity of the Gr-SH and Gr-NH_2_ arising due to the presence of higher density of the modifier molecules which convert more number of sp^2^ carbons of Gr into sp^3^ and/or due to the inaccessibility of the modified surfaces to the probe owing to the presence of higher density of modifier molecules. Similar behavior is also observed with K_4_[Fe(CN)_6_], i.e. the redox response of the probe is completely blocked at Gr-SH and Gr-NH_2_ (shown in Fig. 8C,D). This behavior implies the suppression of redox probe response originating either from the inaccessible layer formed by the higher density of molecules or transformation Gr to either insulating or poorly conducting surface.

**Figure 9:**
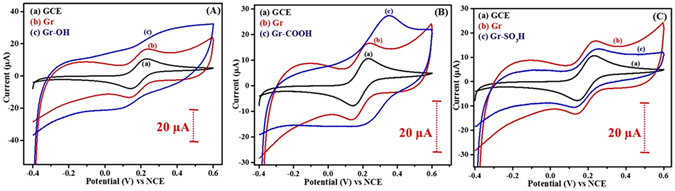
Cyclic voltammograms (a) GCE, (b) Gr, (c) in (**A**) Gr-OH, in (**B**) Gr-COOH and in (**C**) Gr-SO_3_H recorded in 1 M KCl solution containing 1 mM K_4_[Fe(CN)_6_] at 50 mV s^−1^. The dotted bars represent the magnitude of current scale.

As shown in Fig. 9, the ΔE_p_ of anionic redox probe on Gr-OH (265 mV), Gr-COOH (138 mV) and Gr-SO_3_H (123 mV) significantly increased when compared to that of untuned graphene (94 mV). This behavior can be due to the charge based repulsions between the probe and the negatively charged reactive end groups present on the graphene. The peak current and peak potential difference values are tabulated in Table 1 for better understanding.

**Table 1:** The electrochemical parameters of graphene and surface tuned graphenes towards [Ru(NH_3_)_6_]Cl_3_ and K_4_[Fe(CN)_6_]Cl_3_ electro-oxidation.

Surface/parameters	K_4_[Fe(CN)_6_]			[Ru(NH_3_)_6_]Cl_3_		
	I_p_^a^ (µA)	I_p_^c^ (µA)	∆E_p_ (mV)	I_p_^a^ (µA)	I_p_^c^ (µA)	∆E_p_ (mV)
GCE	10.54	9.8	82	9.54	9.68	87
Graphene	10.24	9.96	94	18.57	18.62	78
Gr-OH	11.05	10.33	265	22.68	25.48	74
Gr-COOH	16.06	13.3	138	12.85	13.93	85
Gr-SO_3_H	16.7	13.3	123	13.96	14.33	83
Gr-SH	—	—	—	—	—	—
Gr-NH_2_	—	—	—	—	—	—

**Figure 10:**
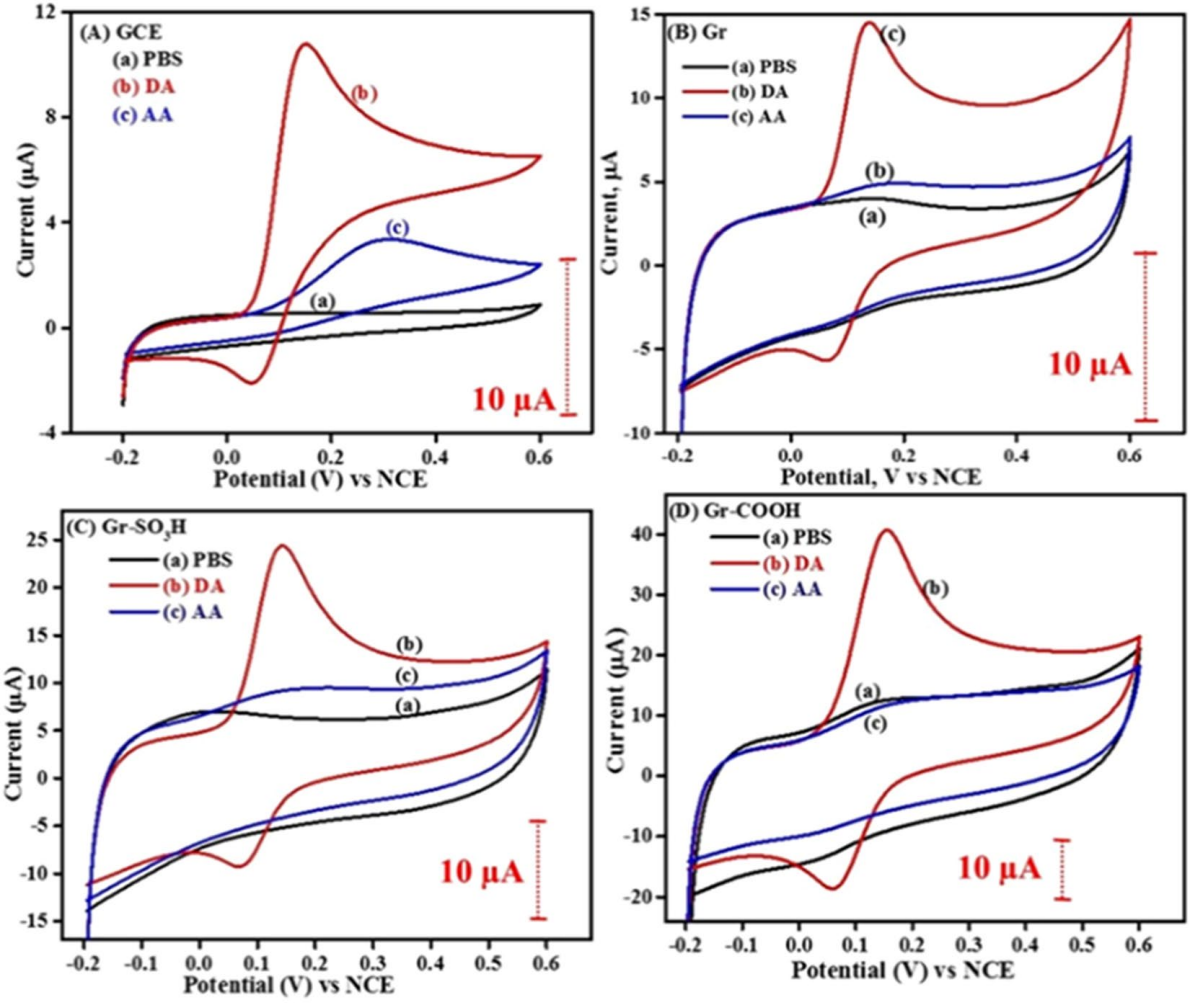
Cyclic voltammograms of (**A**) GCE, (**B**) Gr, (**C**) Gr-SO_3_H and (**D**) Gr-COOH recorded in (a) 0.1 M PBS (pH = 7), (b) 500 µM DA in 0.1 M PBS and (c) 500 µM AA in 0.1 M PBS at 50 mV s^−1^. The dotted bars represent the magnitude of current scale.

**Figure 11:**
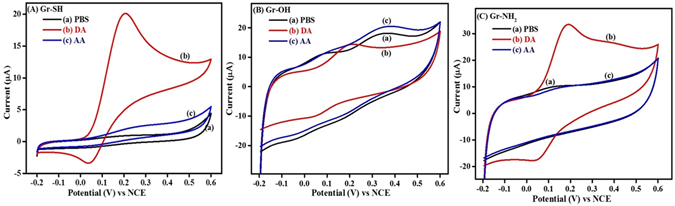
Cyclic voltammograms of (**A**) Gr-SH, (**B**) Gr-OH and (**C**) Gr-NH_2_ recorded in (a) 0.1 M PBS (pH = 7), (b) 500 µM DA in 0.1 M PBS and (c) 500 µM AA in 0.1 M PBS at 50 mV s^−1^.

In addition to the redox probes, two essential bioanalytes dopamine and ascorbic acid are also examined to demonstrate the suitability of the surface tuned graphenes towards the selective detection of dopamine. DA and AA oxidize at similar potentials at bare GCE and Gr as shown in Fig. 10A,B. Decorating an electrode with anionic membrane or polymer or functionality is a conventional practice to discriminate DA from AA by suppressing the response of AA by electrostatic repulsions^32–34^. The same has been realized in the case of Gr-COOH and Gr-SO_3_H in the presence of DA and AA as shown in the Fig. 10C,D. As one can anticipate, the negatively charged carboxylate (-COO^−^) and sulfonate (-SO_3_^−^) functionalities suppressed the response of AA while increasing response of DA by charge-based interactions. Figures 10 and 11 display the cyclic voltammograms of dopamine and ascorbic acid on Gr-SH, Gr-OH, Gr-NH_2_, Gr-COOH, and Gr-SO_3_H. The peak potentials and redox currents obtained are presented in Table 2 for better understanding.

**Table 2:** The electrochemical parameters of graphene and surface tuned graphenes towards DA and AA.

Electrode/parameters	DA					AA	
	I_pa_ (µA)	I_pc_ (µA)	E_pa_ (mV)	E_pc_ (mV)	ΔE_p_ (mV)	I_pa_ (µA)	E_pa_ (mV)
GCE	10.8	2.07	145	48	97	3.34	303
Graphene	14.5	5.3	136	70	66	5	190
Gr-SH	20.1	3.46	204	41	163	—	—
Gr-OH	14.2	10.4	189	51	138	20.59	352
Gr-NH_2_	33.6	17.71	189	42	147	—	—
Gr-COOH	40.7	18.46	150	60	90	—	—
Gr-SO_3_H	24.44	9.26	141	78	63	—	—

From Table 2, it is clear that in the case of dopamine higher redox currents and lower peak to peak potential differences are observed on Gr-COOH and Gr-SO_3_H when compared to the rest. In the case of Gr-COOH and Gr-SO_3_H, the oxidation of DA is kinetically favorable as reflected by their peak to peak separation values, as shown in Fig. 11. The peak to peak separation values of DA are in the order Gr-SO_3_H (63 mV) < Gr-COOH (90 mV) < Gr-OH (138 mV) < Gr-NH_2_ (147 mV) < Gr-SH (163 mV). Hence, this substantiates that the Gr-COOH and Gr-SO_3_H have better electrocatalytic activity towards DA. The redox response of AA is obtained only on Gr-OH, while the others show no or negligible response, which manifests that the reactive end groups except -OH have enough ability to block the AA from reaching the electrode by electrostatic repulsion.

These results together with XPS and FE-SEM analysis confirm the tactical surface tuning of graphene with desired reactive end groups such as -SH, -OH, -NH_2_, -COOH and -SO_3_H by the versatile and rational electrochemical approach. The surface tuned graphenes exhibit terminal group dependent interfacial properties which can be useful for numerous applications in the fields of chemical sensors, biosensors, immunosensors, and DNA sensors. Also, the feasibility of post-functionalization of the reactive end groups of the surface tuned graphenes further springs the applications of graphene in different fields.

## Conclusions

The tactical tuning of the surface and interfacial properties of graphene using different arylamines has been realized by the versatile and rational electrochemical method. The protocol and the functional groups of the surface tuned graphenes are well examined by CV, XPS, and FE-SEM. The interfacial properties of graphene are tactically tuned by tethering the desired reactive end groups (-SH, -OH, -NH_2_, -COOH and -SO_3_H) on graphene. As one can anticipate, the surface tuned graphenes exhibited reactive end group dependent interfacial properties as realized by CV studies with the redox probes as well as bioanalytes. The reported protocol offers a way to understand the fundamental properties and useful applications of the surface tuned graphenes with desired reactive end groups. Besides, the post-functionalization feasibility of reactive end groups of the surface tuned graphenes further springs the scope of usefulness of the thus prepared material. Further research should be directed towards the control of the coverage, the density of the reactive end groups, and the effect of other reactive end groups on the graphene and their impact on the physicochemical and electrochemical properties of the surface tuned graphenes towards the desired chemical and electrochemical applications.

## Electronic supplementary material

Electronic supplementary information
