# Supplementary material for: Tactical tuning of the surface and interfacial properties of graphene: A Versatile and rational electrochemical approach
Source: Sci Rep. 2017 Aug 21;7:8354. doi: 10.1038/s41598-017-08627-1 (PMC5567138; doi:10.1038/s41598-017-08627-1)
Supplement: Supplementary file 1 — Electronic supplementary information [file 41598_2017_8627_MOESM1_ESM.doc]

**Electronic supplementary information**

# **Tactical tuning of the surface and interfacial properties of graphene:**

# **A Versatile and rational electrochemical approach**

# Chiranjeevi Srinivasa Rao Vusa^*#^, Venkatesan Manju^*^, Aneesh. K, Sheela Berchmans^#^, Palaniappan Arumugam^#^

Council of scientific and industrial research- Central electrochemical research institute, Karaikudi-630003


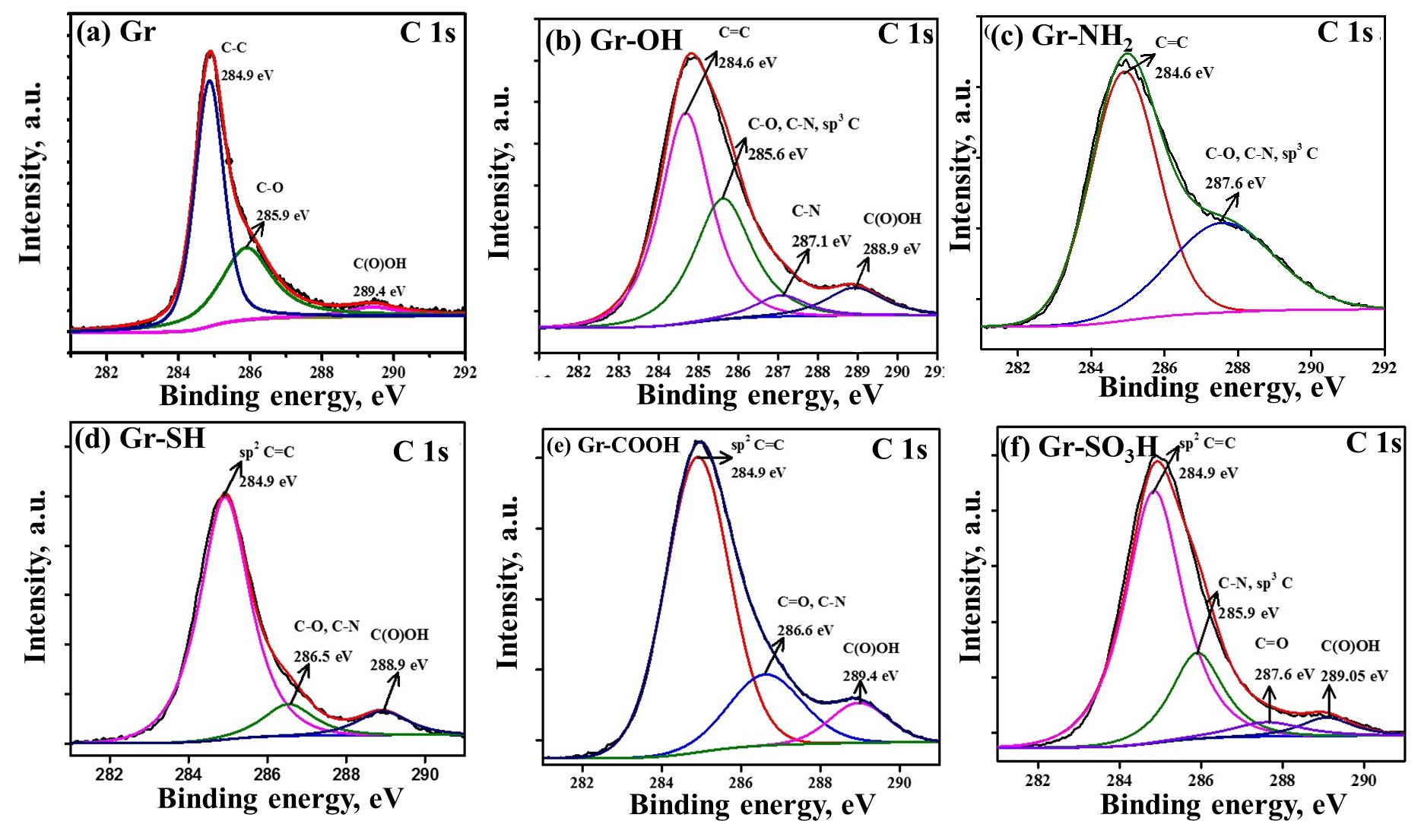


**Figure S1.** The high resolution C 1s XPS spectrum of (a) graphene, (b) hydroxylated graphene, (c) aminated graphene, (d) thiolated graphene, (e) carboxylated graphene and (c) sulfonated graphene.


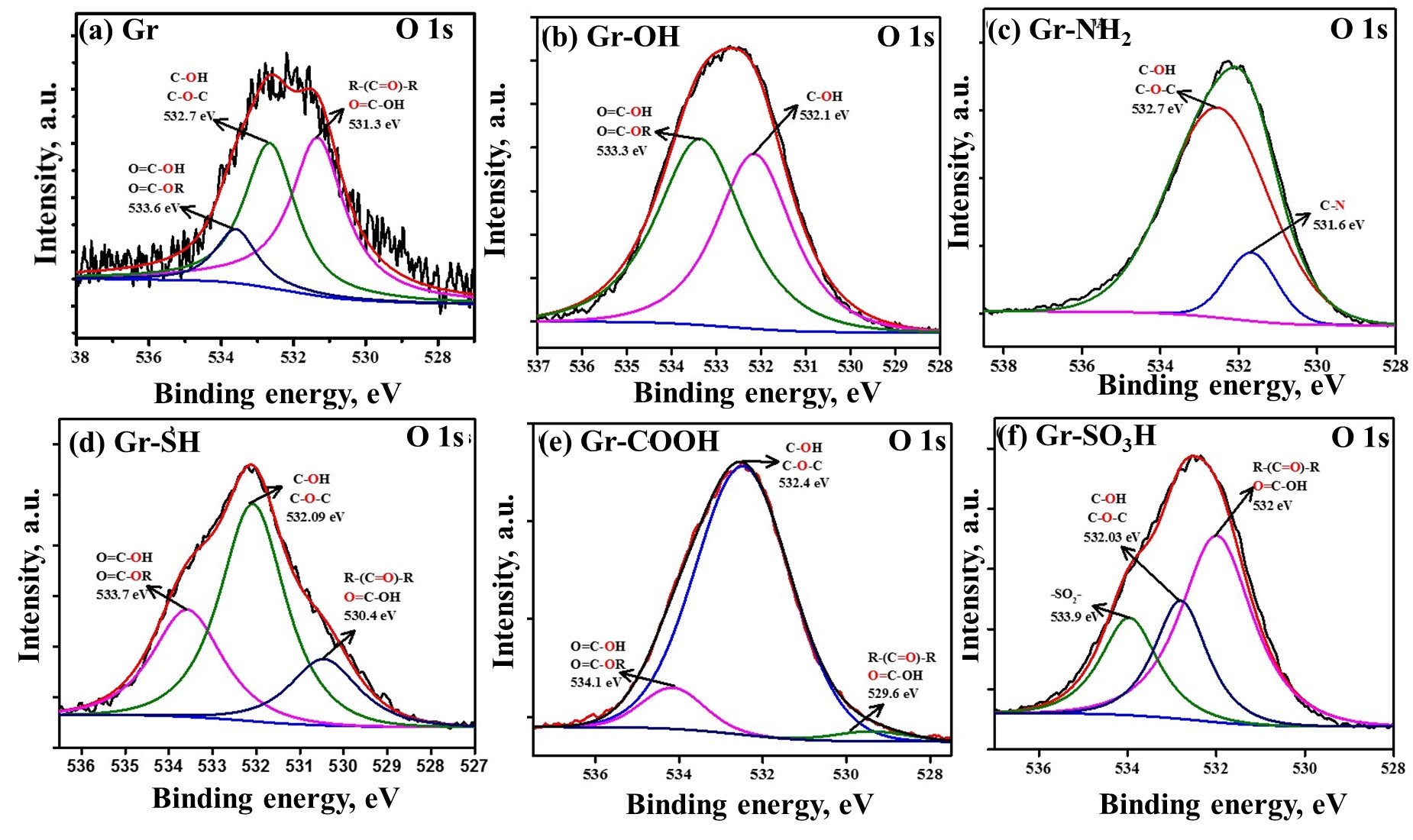


**Figure S2.** The high resolution O 1s XPS spectrum of (a) graphene, (b) hydroxylated graphene, (c) aminated graphene, (d) thiolated graphene, (e) carboxylated graphene and (c) sulfonated graphene.

**Table S1: The surface elemental composition of surface tuned graphenes.**

| **Surface/** | **C 1s** | | **O 1s** | | **N 1s** | | **S 2p** | |
| --- | --- | --- | --- | --- | --- | --- | --- | --- |
| **Parameters** | **Peak BE** | **at. %** | **Peak BE** | **at. %** | **Peak BE** | **at. %** | **Peak BE** | **at. %** |
| **Gr** | 284.6 | 90.38 | 532.6 | 9.62 | - | - | - | - |
| **Gr-OH** | 284.54 | 80.38 | 532.21 | 17.98 | 399.74 | 1.63 | - | - |
| **Gr-NH_2_** | 285.4 | 69.7 | 532.06 | 22.59 | 399.89 | 7.71 | - | - |
| **Gr-SH** | 284.84 | 76.77 | 532.21 | 19.71 | 399.52 | 1.97 | 163.89 | 1.55 |
| **Gr-COOH** | 285.09 | 78.02 | 532.23 | 20.19 | 399.84 | 1.79 | - | - |
| **Gr-SO_3_H** | 284.59 | 73.6 | 532.08 | 13.04 | 399.56 | 5.83 | 163.95 | 6.12 |

**Table S2: The relative distribution of different nitrogen components in the surface tuned graphenes.**

| **Surface/ parameters** | **N 1s** | | | |
| --- | --- | --- | --- | --- |
|  | **Components** | **Peak BE** | **Peak Area** | **% RD** |
| **Gr** | - | - | - | - |
| **Gr-OH** | sp^3^ C-N  sp^2^ C-N | 399.7  400.5 | 168117.38  167918.90 | 50  49.97 |
| **Gr-NH_2_** | sp^3^ C-N  sp^2^ C-N  protonated amine | 399.3  400.01  402.4 | 144128.28  169246.27  145105.04 | 31.4  36.9  31.6 |
| **Gr-SH** | sp^3^ C-N  sp^2^ C-N | 399.2  400.0 | 121614  121860 | 49.9  50 |
| **Gr-COOH** | sp^3^ C-N  sp^2^ C-N  protonated amine | 399.3  400.03  401.2 | 150606.49  150973.30  150019.35 | 33.35  33.43  33.22 |
| **Gr-SO_3_H** | sp^3^ C-N  sp^2^ C-N | 399.1  399.9 | 131951.51  141551.40 | 48.24  51.75 |

RD represents the relative distribution of different species in the sample

**Calculation of electrochemical active surface area of graphene modified GCE**

Randles-Sevcik equation can be used to calculate electrochemical active surface area of graphene and related materials modified electrodes ^12^ as follows.


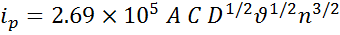
 ………………. (S1)

Where i_p_ is the oxidation peak current of K_4_[Fe(CN)_6_] in ampere obtained from cyclic voltammogram recorded in the solution of 0.5 M KCl containing 5 mM K_4_[Fe(CN)_6_] at the scan rate of 50 mVs^-1^, A is the electrochemical active surface area of the graphene-modified GCE in cm^2^, C is the Concentration of the analyte (5 mM K_4_[Fe(CN)_6_]) in mol cm^-3^, D is the Diffusion coefficient of K_4_[Fe(CN)_6_] reported as 7.62
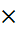
10^-6^ cm^2^ s^-1^, ʋ is the scan rate in V s^-1^ and n is the number of electrons transferred in the redox reaction (usually 1).

The electrochemical active surface area A was calculated using I vs. ʋ^1/2^ plot as follows. Cyclic voltammograms were recorded at various scan rates from 10 to 180 mV under the above experimental conditions and the corresponding I vs. ʋ^1/2^ plot was constructed to calculate the A form the slope of the I vs. ʋ^1/2^ plot.


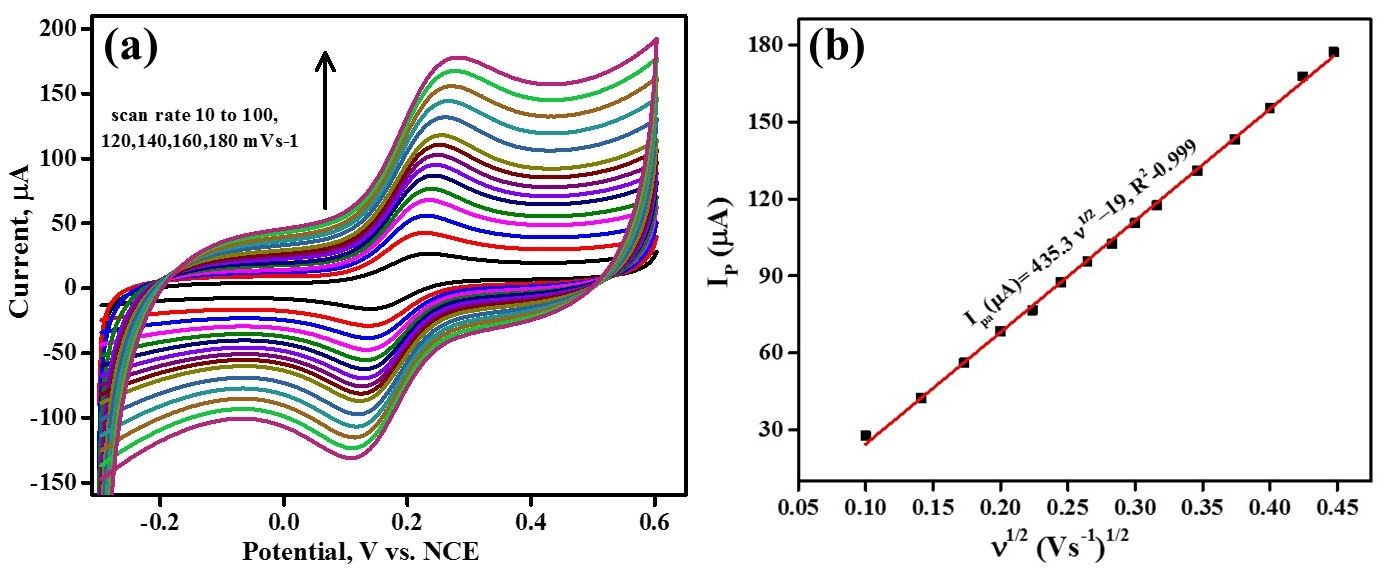


**Figure S3:** Cyclic voltammograms of Gr modified GCE in the solution of 0.5 M KCl containing 5 mM K_4_[Fe(CN)_6_] at the scan rate of 50 mVs^-1^ (a) and the corresponding I vs. ʋ^1/2^ plot (b).

The linear regression equation observed for plot I vs. ʋ^1/2^ with R^2^-0.999 is shown in Figure S3 b. From this,


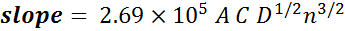


Hence, the area of the Gr modified GCE


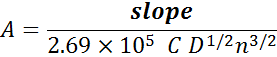


By substituting the slope, C, D, and n in the above equation, the area is calculated to be 0.117 cm^2^. The value of area used herein the manuscript for all the calculations is 0.1104 cm^2^ which is an average value of three independent experiments done with three different Gr modified GCEs under optimized conditions.
